# Key Roles of Hyaluronan and Its CD44 Receptor in the Stemness and Survival of Cancer Stem Cells

**DOI:** 10.3389/fonc.2015.00180

**Published:** 2015-08-10

**Authors:** Theerawut Chanmee, Pawared Ontong, Koji Kimata, Naoki Itano

**Affiliations:** ^1^Department of Molecular Biosciences, Faculty of Life Sciences, Kyoto Sangyo University, Kyoto, Japan; ^2^Biotechnology, Division of Engineering, Graduate School of Engineering, Kyoto Sangyo University, Kyoto, Japan; ^3^Research Complex for Medical Frontiers, Aichi Medical University, Nagakute, Japan; ^4^Institute of Advanced Technology, Kyoto Sangyo University, Kyoto, Japan

**Keywords:** hyaluronan, CD44, epithelial-to-mesenchymal transition, cancer stem cell, multidrug resistance

## Abstract

Cancer stem cells (CSCs) represent a unique subpopulation of self-renewing oncogenic cells that drive cancer initiation and progression. CSCs often acquire multidrug and oxidative stress resistance and are thereby thought to be responsible for tumor recurrence following treatment and remission. Although the mechanisms responsible for CSC generation, maintenance, and expansion have become a major focus in cancer research, the molecular characteristics of CSCs remain poorly understood. The stemness and subsequent expansion of CSCs are believed to be highly influenced by changes in microenvironmental signals as well as genetic and epigenetic alterations. Hyaluronan (HA), a major component of the extracellular matrix, has recently been demonstrated to provide a favorable microenvironment for the self-renewal and maintenance of stem cells. HA directly and indirectly affects CSC self-renewal by influencing the behavior of both cancer and stromal cells. For instance, HA in the tumor microenvironment modulates the function of tumor-associated macrophages to support CSC self-renewal, and excessive HA production promotes the acquisition of CSC signatures through epithelial-to-mesenchymal transition. The importance of HA in mediating CSC self-renewal has been strengthened by the finding that interactions between HA and its receptor, CD44, propagate the stemness of CSCs. HA–CD44 interactions evoke a wide range of signals required for CSC self-renewal and maintenance. CD44 also plays a critical role in the preservation and multidrug resistance (MDR) of CSCs by transmitting survival and anti-apoptotic signals. Thus, a better understanding of the molecular mechanisms involved in HA and CD44 control of CSC stemness may help in the design of more effective therapies for cancer patients. In this review, we address the key roles of HA and CD44 in CSC self-renewal and maintenance. We also discuss the involvement of CD44 in the oxidative stress and MDR of CSCs.

## Introduction

Cancer stem cells (CSCs) are a subpopulation of oncogenic cells that have an ability to self-renew and the potential for generating heterogeneous malignant progenies (1, 2). CSCs are thought to be responsible for cancer initiation, progression, and metastasis. Furthermore, they can acquire resistance to chemotherapy and oxidative stress. The CSC hypothesis has therefore garnered much attention due to the high-therapeutic potential of CSC-targeting treatments. Recently, a great amount of effort has been invested on identifying CSCs in various types of cancers and the key molecules involved in controlling the unique properties of CSC populations. Putative markers specific for CSCs have been discovered and enable us to distinguish CSCs from normal stem cells and non-stem cancer cells (Table 1). Although there is still debate on the origin of CSCs, several prominent studies have contributed important insights on the generation of these cells. CSCs might appear after sequential mutations in specific stem cells or early cell progenitors. Phenotypic plasticity has also been demonstrated to allow cancer cells to dynamically enter into a stem-cell state (3). Furthermore, emerging evidence has suggested that cancer cells undergoing epithelial-to-mesenchymal transition (EMT) acquire stem-like cell signatures, such as the ability of self-renewal (4). The stemness properties and subsequent expansion of CSCs are highly influenced by microenvironmental signals as well as genetic and epigenetic regulation (5, 6). Among such microenvironmental molecules, hyaluronan (HA) has recently been shown to maintain the stemness of normal stem cells. HA could also promote the acquisition of CSC signatures through EMT and support CSC self-renewal by modulating the function of stromal cells. CD44 is a known HA receptor and prominent marker of CSCs in several types of cancer (7–9). Early observations have demonstrated a significant correlation between CD44 and tumor mortality (10), and growing evidence has suggested that CD44 is not only a CSC marker but also a mediator of many functional roles of CSCs. Collectively, both HA and CD44 appear to be strongly linked to the generation, maintenance, and survival of CSCs. This review focuses on the key roles of HA and CD44 in the regulation of CSC stemness and sustainability.

**Table 1:** **Cell surface markers of cancer stem cells**.

CSC marker(s)	Cancer type	Reference
CD44^+^	Head and neck, gastric, lung	(11–13)
CD44^+^CD24^+^	Head and neck	(14)
CD44^+^CD24^−^	Breast	(15, 16)
CD44^+^CD24^+^ESA^+^	Pancreatic	(17)
CD44v3^high^ALDH1	Head and neck	(18)
CD44^+^α_2_β_1_^high^CD133^+^	Prostate	(9)
CD44^+^EpCAM^high^	Colorectal	(8)
CD44^+^CK5^+^CK20^−^	Bladder	(19)
CD44^+^CD117^+^	Ovarian	(20)
CD44^+^CD90^+^	Lung, Liver	(21, 22)
CD34^+^CD38^−^	Acute myeloid leukemia, acute lymphoid leukemia	(23)
CD133	Brain, colon, ovary, lung, melanoma	(24–28)
ABCB5^+^	Melanoma	(29)

## HA and its CD44 Receptor

**Figure 1:**
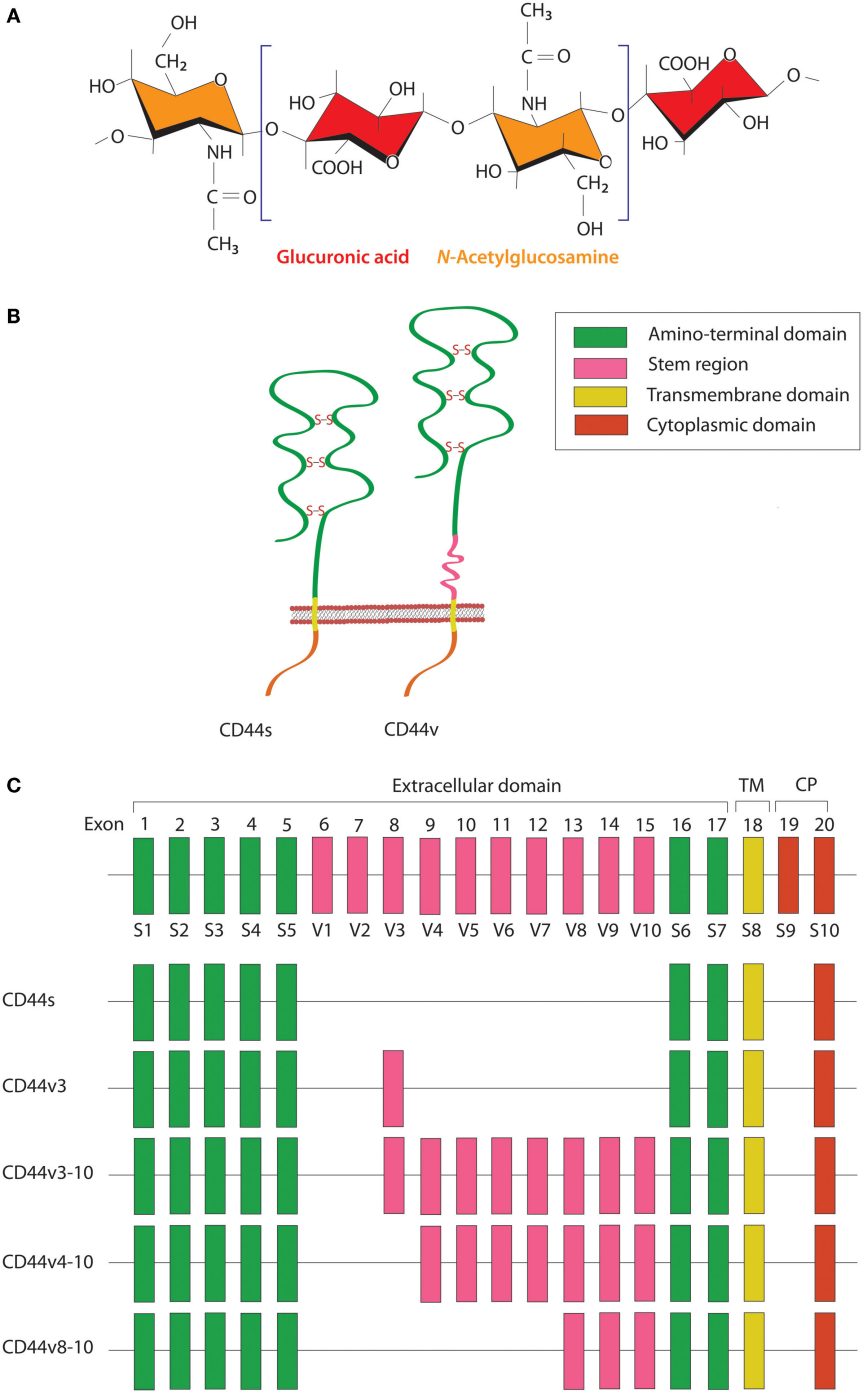
**Schematic diagram of HA and CD44 structures**. **(A)** HA is a linear polysaccharide composed of repeating disaccharide units of glucuronic acid and *N*-acetylglucosamine. **(B)** The CD44 protein consists of an N-terminal HA-binding link-homology module, stem region, transmembrane domain, and short C-terminal cytoplasmic domain. **(C)** CD44 is encoded by 20 exons, among which exons 1–5 and 16–18 are constant regions contained in all CD44 isoforms. Exon 19 encodes an alternatively spliced short form that spliced out in most CD44 isoforms. Exons 6–15 are variant exons, typically identified as vl–v10, which can be alternatively spliced and give rise to multiple variant CD44. In human, exon 6 (exon v1) encodes a stop codon and is not expressed. CD44 variant can contain one or more variant regions, such as CD44v3, CD44v3–10, CD44v4–10, or CD44v8–10.

HA is a linear polysaccharide that belongs to the glycosaminoglycan family. It is composed of repeating disaccharide units of glucuronic acid and *N*-acetylglucosamine (Figure 1A) and has a molecular mass ranging from 10^3^ to 10^7^ Da, depending on tissue type and physiological condition. HA is ubiquitously found in the extracellular matrix (ECM) of vertebrate tissues and is known to contribute to cell proliferation, migration, and adhesion (30). A wide variety of HA-binding molecules also participate in the assembly of pericellular HA–ECM to regulate HA function. Despite its relatively simple chemical composition, HA mediates many important processes during embryonic morphogenesis, tissue regeneration, and wound healing (31). Abnormalities in HA metabolism have been implicated in numerous conditions, including inflammatory disorders, cardiovascular diseases, and cancer. The biosynthesis and degradation processes of HA are tightly regulated by three HA synthases (HAS1–3) and several hyaluronidases, respectively. HAS possesses a glycosyltransferase ability that catalyzes the polymerization of glucuronic acid and *N*-acetylglucosamine to the nascent HA chain. Each HAS isoform exhibits different enzymatic properties with regard to stability, enzyme kinetics, and rate of chain elongation (32).

CD44 is a transmembrane glycoprotein known to act as a receptor for a wide variety of ECM ligands, such as HA, collagen, fibronectin, laminin, osteopontin, and heparin-binding epidermal growth factor (HB-EGF). Structurally, CD44 proteins contain an N-terminal HA-binding link-homology module, stem region, transmembrane domain, and short C-terminal cytoplasmic domain (Figure 1B). The receptor binds to its major physiologic ligand, HA, via the N-terminal HA-binding link-homology module. CD44 can exerts an intracellular signaling by associated with its ligand or modifying the function of growth factor receptors and thereby affected to the downstream signaling molecules through the cytoplasmic tail. The CD44 cytoplasmic tail can form complex with many cytoskeletal proteins and intracellular signaling components, such as Rho-family GTPases, Src family kinases, and associated molecules that affected to cell adhesion and migration (33). The active and inactive states of CD44 are multiply regulated by dimerization and post-translational modification, such as *N*- and *O*-glycosylation, under appropriate circumstances (34, 35). CD44 is encoded by a single gene containing 20 exons. The standard isoform (CD44s) is encoded by constant exons 1–5 and 16–20 and translated into a polypeptide of a molecular mass of 80–85 kDa. The variable exons 6–15 (v1–v10) are regulated by alternative splicing to generate multiple variant isoforms (Figure 1C) that are expressed in tissue- and stage-specific manners during both embryonic development and carcinogenesis (36). Most notably, CD44 is a common marker of CSCs in several types of human carcinoma, including cancers of the breast, prostate, bladder, colon, ovary, stomach, cervix, uterus, nasopharynx, head and neck, lung, and pancreas (Table 1).

## Impact of HA and CD44 on Cancer Progression

Elevated levels of HA are found in many types of human cancer, and HA accumulation within tumor stroma has been shown to be closely correlated with poor prognosis and survival in cancer patients (37–39). Clinicopathological studies have indicated that the high HA levels in the peri- and intratumoral stroma of epithelial ovarian cancer were significantly associated with poor differentiation, progressed histological type, and advanced stage (37). High stromal HA was also strongly related to reduced overall and disease-free survival and was more frequently observed in metastatic lesions than in primary tumors (38). HAS overexpression has similarly been implicated in cancer progression (40). In human breast cancer, localization and elevated expression of HAS were correlated with prognostic factors and patient outcome (41). Specifically, HAS1 expression in breast carcinoma cells associated with a high relapse rate and short overall survival, while expression levels of stromal HAS1 and HAS2 were positively related to tumor size and lymph node metastasis (41). Elevated HAS2 expression was also witnessed in highly invasive breast carcinoma cells (42). Experimental evidence has highlighted the functional roles of HA in tumor growth and metastasis. For instance, our previous study demonstrated that mutant mammary carcinoma cells lacking the ability to synthesize HA displayed a significant decrease in metastatic ability in an experimental model of lung metastasis, which was restored by the re-establishment of HA synthesis by HAS1 transfection in accordance with formation of an HA-rich pericellular coat (43). Kosaki et al. genetically manipulated HA production by HAS2 transfection in a human HT1080 fibrosarcoma cell line and observed the enhancement of anchorage-independent growth and tumorigenicity (44). Similarly, forced expression of HAS3 promoted the tumorigenic ability of melanoma cells by accelerating tumor angiogenesis (45), but suppression of HAS2 or HAS3 inhibited the initiation and progression of primary and secondary tumor formation *in vivo* (46). Thus, HA appears to be a crucial factor in determining the malignant characteristics of cancers. The functional importance of HA in tumor progression was also demonstrated in our recent study using an HA-overproducing breast cancer model in Has2 transgenic mice. We found that HA overproduction in cancer cells promoted tumor progression by accelerating angiogenesis and lymphangiogenesis concurrently with the formation of stromal structures (40, 47).

The HA-rich tumor microenvironment contributes prominently to tumor neovascularization by enhancing the recruitment of host stromal cells, such as fibroblasts, monocytes/macrophages, endothelial cells, and mesenchymal stem cells. The pericellular HA matrix with cable-like structure was reported to facilitate monocyte/macrophage recruitment in vascular lesions via CD44 (48), which, in turn, released pro-angiogenic factors essential for tumor angiogenesis (49). Notably, macrophages were seen to preferentially traffic to HA-enriched stromal structures in spontaneous mammary tumors (47). Depletion of macrophages reduced both tumor angiogenesis and lymphangiogenesis in HA-producing tumors, and disruption of the *Has2* gene in tumor-associated fibroblasts (TAFs) led to impairment of macrophage infiltration following inoculation of mammary carcinoma cells into nude mice. Concurrently with diminished macrophage recruitment, ­Has2-null TAFs attenuated tumor angiogenesis and lymphangiogenesis. These findings strongly suggest that stromal HA promotes tumor neovascularization via the preferential engagement of tumor-associated macrophages (TAMs).

HA is typically present in normal tissues as high-molecular weight HA (HMW-HA) with an average molecular weight of approximately 10^7^ Da. The appearance of its lower molecular weight could influence cell behavior. Significant amounts of HA fragments have been detected in malignant tumors, for which increases correlated with tumor aggressiveness in humans (50–52). The properties and functions of HA appear to depend on molecular size; HA oligosaccharides of 4–25 disaccharide units exhibited pro-angiogenic capabilities by enhancing the proliferation and migration of endothelial cells, while HMW–HA was observed to function as an anti-angiogenic agent (53, 54). Dependent on polymer length, HA can also exist as both a pro- and an anti-inflammatory molecule *in vivo* (55, 56). These contradictory functions of HA in angiogenic and inflammatory processes are regulated by a complex interplay between HA production and degradation.

Interactions between HA and CD44 have been shown to play essential roles in tumor cell growth, survival, migration, and metastasis. Inhibition of HA–CD44 binding by HA oligosaccharides led to suppression of tumor cell growth *in vivo* by disruption of the phosphoinositide 3-kinase (PI3K)/Akt survival pathway (57). Knockdown of CD44 decreased the adhesiveness of human colon cancer cells to HA, cancer colony-forming ability in soft agar assays, and xenograft tumorigenicity, while increasing susceptibility to etoposide-induced apoptosis (58). CD44 expression also correlated with tumor recurrence after surgical treatment (59–61). Activation of CD44 has been seen to promote multidrug resistance (MDR) in cancer cells, which is a hallmark of therapeutic resistance. Not surprisingly, neutralizing antibodies targeting CD44 effectively inhibited tumor growth and prevented tumor relapse after chemotherapy-induced remission in an orthotopic xenograft model of human breast cancer (62).

Expression of CD44 variant proteins has also been implicated in tumor progression (63). The CD44v3 isoform was observed to play a role in the progression of head and neck squamous cell carcinoma (HNSCC) by stimulating cell growth, migration, and metalloproteinase (MMP) expression (64). Transfection of this isoform into an HNSCC cell line markedly increased tumor cell migration (64). Moreover, overexpression of specific CD44 variants in non-metastasizing pancreatic carcinoma and mammary adenocarcinoma cell lines led to metastatic behavior (65). All of these findings highlight the important roles of CD44 in cancer progression and recurrence.

## HA and EMT

**Figure 2:**
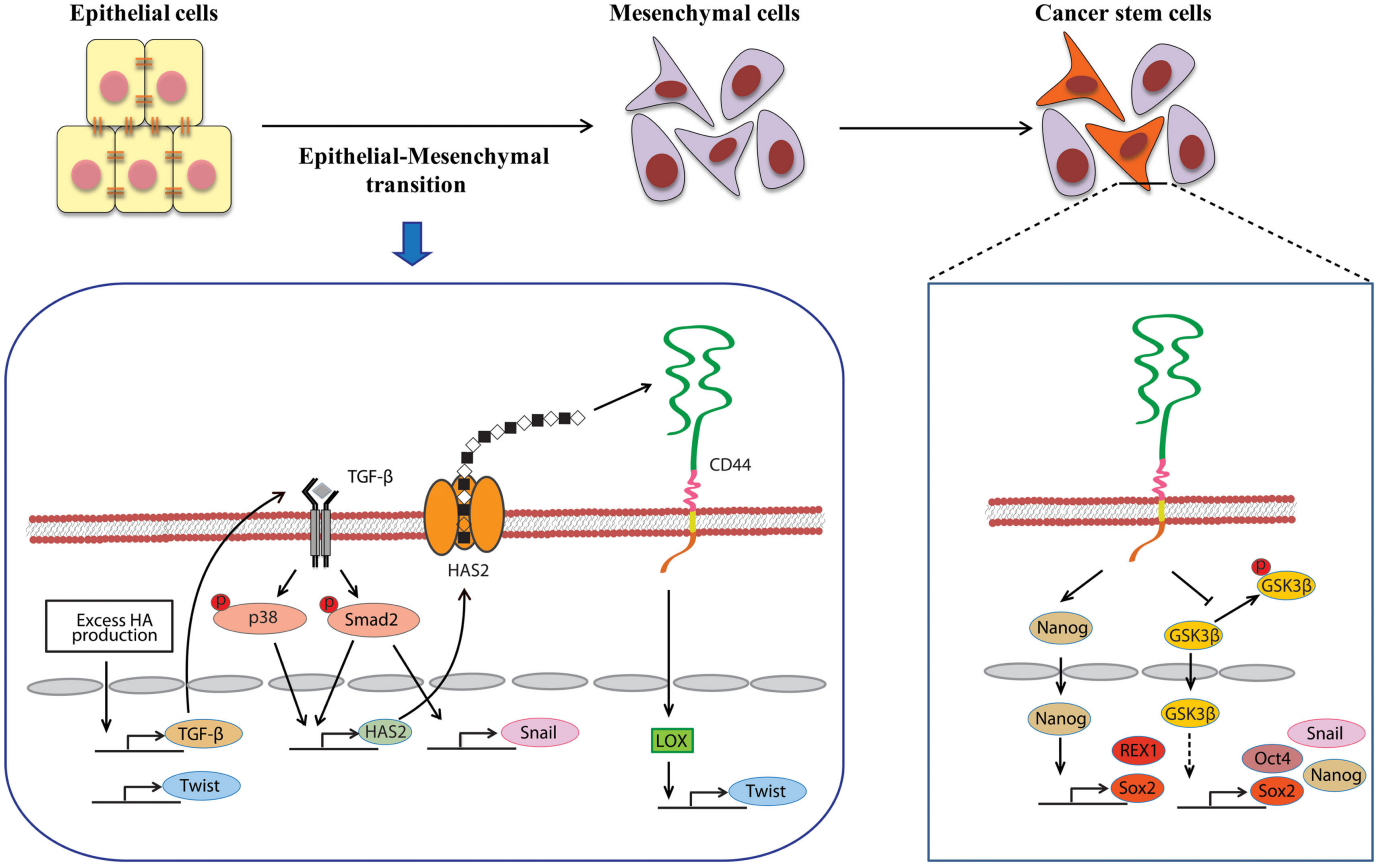
**Involvement of HA in the induction of EMT and CSC self-renewal**. An alternative mechanism of HA–CD44 interaction-promoted EMT relying on activation of the lysyl oxidase promoter leads to the up-regulation of Twist. HA overproduction also induces EMT through the coordinated regulation of Twist and TGF-β-Snail signaling. In addition, TGF-β facilitates EMT by increasing the expression of HAS2. This stimulatory effect requires the activation of Smad and p38 MAPK. CD44 maintains the self-renewal ability of CSCs through Nanog and GSK3β. HA and CD44 promote Nanog protein association with CD44 followed by activation of the stem-cell regulators Rex1 and Sox2. CD44 also inhibits the phosphorylation of GSK3β to maintain the functional indicators of cancer stemness, including EMT and stem-cell markers (e.g., Oct4, Sox2, and Nanog).

A close link between EMT and CSC properties has emerged to enable a greater understanding of the molecular mechanisms underlying the expansion and maintenance of CSCs in tumor masses. Increasing evidence suggests that HA plays a central role in EMT during embryonic development and malignant progression. Zoltan-Jones et al. demonstrated that forced HAS2 expression induced Madin–Darby canine kidney and MCF-10A human mammary epithelial cells to acquire mesenchymal-like phenotype as defined by a down-regulation of E-cadherin at intercellular boundaries, up-regulation of vimentin, and fibroblast-like morphology (66). Ectopic expression of murine Has2 in non-HA-producing mesothelioma cells also invoked conversion to fibroblastic morphology (67). *In vivo* experiments have confirmed the importance of HA in the induction of EMT. Disruption of the *Has2* gene abrogated normal cardiac morphogenesis and HA-mediated transformation of epithelium to mesenchyme (68). We produced conditional transgenic mice carrying the murine *Has2* gene and observed that HA overproduction in spontaneous mammary tumors resulted in a loss of epithelial phenotype in tumor cells by down-regulation of E-cadherin and induced nuclear translocation of β-catenin, both of which are hallmarks of EMT (40). Transcriptional networks that drive EMT (e.g., Snail, Twist, and Zeb1) have also been found to dynamically change during HA-induced EMT. We witnessed that HA overproduction up-regulated Snail and Twist expression in a mammary carcinoma cell (Figure 2) (69). Furthermore, HA association with CD44 was suggested to induce Twist expression following CD44 nuclear translocation and activation of the lysyl oxidase promoter in human breast cancer (Figure 2) (70).

During malignancy progression, activated fibroblasts, immune cells, and other stromal cells infiltrate into tumors and construct tumor microenvironments through secretion of cytokines and growth factors. The EMT program in tumor cells is extensively regulated by networks of such mediators. Several studies support the functional role of autocrine/paracrine cytokine loops in controlling HA-induced EMT. TGF-β, a known potent EMT inducer that is abundantly secreted by both stromal and tumor cells, has been shown to induce Twist, Snail, and Zeb1 in many types of cancers. Porsch et al. demonstrated that the regulatory mechanisms of TGF-β-induced EMT required HAS2 expression in mammary epithelial cells (Figure 2). Silencing of HAS2 suppressed TGF-β-induced EMT as evidenced by a reduction in the EMT markers, such as fibronectin, Snail, and Zeb1 (71). The release of hepatocyte growth factor (HGF) by stromal cells can also contribute to EMT induction. HGF promoted EMT-like phenotype in hepatocellular carcinoma cells through the down-regulation of E-cadherin and up-regulation of Snail and N-cadherin (72). Zoltan-Jones et al. showed that elevated HA production evoked mesenchymal and transformed properties in epithelial cells via the transforming activities of HGF (66).

## Roles of HA and CD44 in CSC Self-Renewal and Maintenance

Stem cells reside in a special microenvironmental niche that provides the major cues for promoting survival and maintenance, and HA is a primary ECM component of this stem-cell niche. A recent study indicated that human bone marrow mesenchymal stem cells synthesized a large amount of HA to retain their pericellular coats and maintain their stemness (73). Like in a normal stem-cell niche, HA-rich ECM provides a favorable microenvironment for self-renewal and maintenance of CSCs. HA in the tumor microenvironment also indirectly affects CSC self-renewal by influencing the behavior of stromal cells. Okuda et al. reported that HA produced by metastatic breast cancers promoted the interactions between TAMs and CSCs that preceded platelet-derived growth factor (PDGF)-BB secretion from TAMs. PDGF-BB then activated fibroblasts and osteoblasts to support CSC self-renewal through the induction of fibroblast growth factor (FGF)-7 and FGF-9 expression (74).

Gene expression profiles in cancer cells have suggested an important molecular link between EMT and cancer stemness. Tumor cells undergoing EMT often acquire stem-like cell profiles, such as self-renewing ability. Overexpression of the EMT-related transcription factors, Snail and Twist, induced mesenchymal traits in breast cancer cells and generated subpopulations with a CD44^high^CD24^low^ phenotype that have been observed in human breast CSCs (4, 15). Considerable evidence supporting the key role of HA in EMT induction has surfaced in recent years from cell and animal studies. Forced expression of Has2 resulted in the loss of epithelial phenotype in mammary epithelial cells (66). Our *in vivo* study with a conditional transgenic mouse model allowing Has2 overexpression in breast cancer demonstrated that HA overproduction caused rapid development of aggressive breast carcinoma at a high incidence (40). In the HA-rich carcinoma mass, the cancer cells had acquired an EMT phenotype as defined by the down-regulation of E-cadherin and increased nuclear translocation of β-catenin. Interestingly, HA-overproducing tumor cells acquired stemness via the up-regulation of TGF-β and induction of Snail and Twist, while a loss of EMT by inhibition of TGF-β-Snail signaling or Twist knockdown markedly reduced CSC subpopulations (69). Thus, HA appears to play a crucial role in the acquisition of CSC signatures by promoting EMT.

Several studies have recently highlighted the essentiality of CD44 in CSC self-renewal and maintenance. In HNSCC, ALDH^high^/CD44^high^ cells exhibited similar self-renewal and tumor-initiating properties as CSCs through the suppression of BMP signaling (75). Although its precise mechanism remains undefined, CD44 may act as an intracellular scaffold protein for Smad1, and their interactions may potentiate the long-term survival of CSCs by regulating BMP/Smad signaling (76). Shigeishi et al. have demonstrated that CD44-mediated GSK3β activation is required for the maintenance of CSC properties, whereby CSCs undergoing EMT are dependent on GSK3β activity for mesenchymal phenotype (Figure 2) (77). CD44 knockdown in CSCs resulted in inactivation of GSK3β, induction of epithelial phenotype, and a significant decrease in the number of tumor spheres.

**Figure 3:**
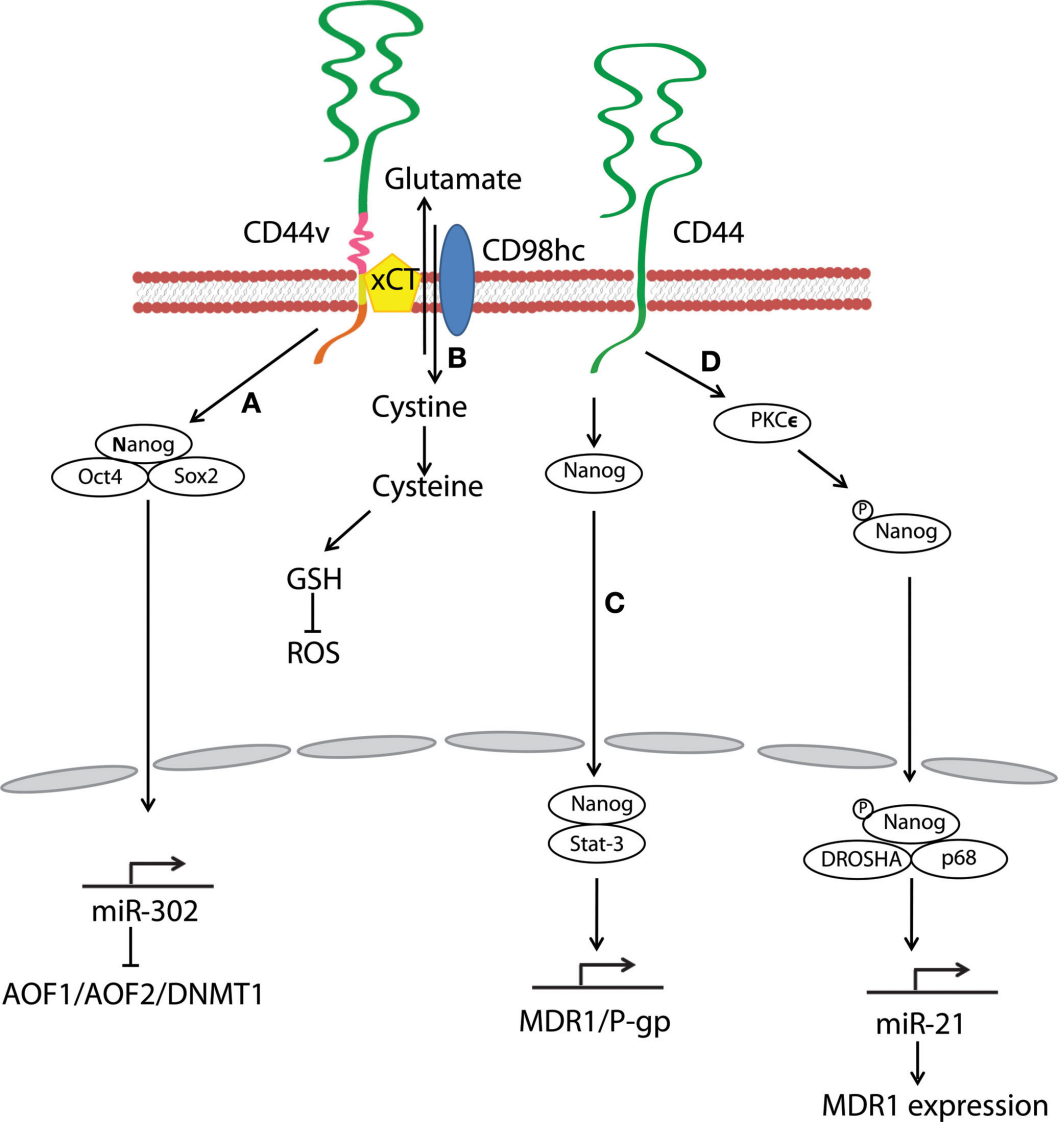
**Hyaluronan and CD44 interactions regulate survival and multidrug resistance in cancer cells**. **(A)** HA–CD44v3 interactions promote CSC signatures in human HNSCC through the formation of stem-cell-specific transcription factor complexes that include Oct4, Sox2, and Nanog. **(B)** A CD44 variant isoform (CD44v8–10) plays a role in stabilizing the glutamate–cystine transporter xCT, which promotes cystine uptake for GSH synthesis and leads to ROS suppression. **(C)** HA–CD44 binding induces interaction of Nanog with Stat-3, which in turn stimulates Stat-3-dependent *MDR1* gene expression. **(D)** The binding of HA to CD44 promotes PKCϵ activity and causes Nanog phosphorylation. Phosphorylated Nanog then translocates from the cytosol to the nucleus and interacts with the microprocessor complex containing RNase III (DROSHA) and RNA helicase (p68) to produce the miR-21 required for up-regulation of MDR1.

Stem-cell-specific transcription factors (e.g., Sox2, Klf4, Oct4, and Nanog) are frequently encountered in human cancers, and their transcriptional networks are necessary for the development and maintenance of cancer stem-like cells (78–80). Interactions between HA and CD44v3 propagated CSC stemness through formation of complexes containing the cell-specific transcription factors Oct4, Sox2, and Nanog in human HNSCC (18). Complex formation stimulated microRNA-302 (miR302) expression and led to the suppression of the epigenetic regulators AOF1/AOF2/DNMT1 and up-regulation of several survival proteins (Figure 3) (18). In addition, HA/CD44-mediated Nanog activation promoted the expression of the stem-cell regulators, such as Rex1 and Sox2 (Figure 2), whereas CD44 knockdown attenuated the expression of stem-cell markers, including Oct4, Nanog, and Sox2 (77). These lines of evidence emphasize that HA and CD44 are indispensable factors in CSC self-renewal, clonal formation, and chemotherapy resistance (81).

## Survival and Anti-Apoptotic Signals Generated by HA–CD44 Interactions

CD44 plays a key role in the continuity of many tumor cells by transmitting survival and anti-apoptotic signals. CD44 and receptor tyrosine kinases (RTKs) have been shown to promote cell survival in a cooperative fashion. HA–CD44 interactions trigger activation of CD44-associated Src, ezrin phosphorylation, and PI3K activation. This signaling cascade subsequently up-regulates the assembly of protein complexes, such as heat shock protein 70 and the co-chaperone CDC37, leading to the phosphorylation and activation of ERBB2/ERBB3 RTKs (82). Besides the induction of survival signals, CD44–RTK cooperation conferred apoptosis resistance via stimulation of cyclooxygenase 2 expression followed by the production of prostaglandin E2 (83). CD44 also prevents apoptosis through molecular alterations in pro- and anti-apoptotic factors. Lakshman et al. demonstrated that the acquisition of resistance to etoposide-induced apoptosis in CD44-expressing colon cancer cells took place concurrently with alterations in caspase 9, caspase 3, Bcl-xL, and Bak (84). Recent studies have provided further evidence for the above notion. In CD44-knockdown colon carcinoma cells, increased apoptosis was accompanied by both reductions in the levels of anti-apoptotic Bcl-2 and Bcl-xL expression and increases in the expression of pro-apoptotic Bax and cleaved caspase 3, caspase 8, and caspase 9 (85). In chronic lymphocytic leukemia cells exhibiting high levels of cell surface CD44, the engagement of CD44 by HA activated the PI3K/Akt and MAPK pathways to induce the expression of anti-apoptotic proteins and, in turn, promote survival (86).

In addition to HA–CD44 interactions, several survival/anti-apoptotic mechanisms have been proposed for apoptosis resistance via CD44 variant isoforms. Binding of CD44v6 to HGF-initiated c-Met signaling that required association of the CD44 cytoplasmic tail with ezrin/radixin/moesin family members to regulate MEK and Erk signaling pathways (87). Moreover, CD44v6 promoted apoptosis resistance through the assembly of ECM that triggered activation of PI3K/Akt and MAPK signaling cascades followed by inactivation of pro-apoptotic Bcl-2-associated death promoter (BAD) (88). Meanwhile, CD44v3 recruited the HB–EGF precursor and a proteolytically active form of matrilysin/MMP7 to the cell surface complex. The HB–EGF precursor was then cleaved by means of CD44-associated MMP7 and the resulting mature HB–EGF engaged and activated anti-apoptotic signals generated by ERBB4 RTK (89). The above findings point to CD44 playing important roles in the survival of cancer cells and CSCs in both HA-dependent and HA-independent manners.

## Drug and Stress Resistance Induced by HA–CD44 Interactions

### Multidrug resistance

Multidrug resistance in CSCs is a major obstacle for effective cancer therapy. CSC drug resistance can be partially attributed to the induction of survival/anti-apoptotic signals, as discussed above. Another hindrance to chemotherapy is rapid drug evacuation from cancer cells mediated by multidrug transporters and MDR genes. Bourguignon et al. demonstrated that HA–CD44 interactions-induced association of Nanog and Stat-3 to stimulate Stat-3-dependent *MDR1* gene expression. Upon HA binding, CD44 also enhanced ankyrin-regulated multidrug efflux (81). They further discovered a novel signaling pathway in which HA-CD44 interactions promoted activation of protein kinase Cϵ followed by Nanog phosphorylation and its nuclear translocation. This cascade eventually resulted in the up-regulation of MDR1 (90). HA–CD44 interactions not only induce MDR1 expression but also up-regulate multidrug resistance protein 2 in non-small cell lung cancer cells (91). Taken together, CD44 figures prominently in the MDR of cancer cells and CSCs via HA binding (Figure 3).

### Resistance to oxidative stress

Oxidative stress caused by an imbalance between reactive oxygen species (ROS) and antioxidants induces cell damage and death. A metabolic switch from oxidative phosphorylation to glycolysis decreases mitochondrial oxygen consumption and ROS levels, and most cancer cells predominantly produce energy by glycolysis, which reduces ROS production. Above all, CSCs effectively gain a survival/growth advantage by adapting their metabolism and maintaining cellular redox homeostasis, even under oxidative stress conditions. Multiple mechanisms explaining how CD44 confers resistance to oxidative stress in CSCs have been proposed. For example, metabolic modulation by CD44 has been demonstrated to contribute to antioxidant status in cancer cells. Tamada et al. found that CD44 interacted with pyruvate kinase M2 and enhanced glycolysis in p53-deficient or hypoxic cancer cells (92); CD44 knockdown then switched the metabolic flux from glycolysis to mitochondrial respiration and increased intracellular levels of ROS. CD44 also controls ROS defense by altering cellular glutathione (GSH) synthesis. Ishimoto et al. described that a CD44 variant isoform (CD44v8–10) regulated redox status in cancer cells by stabilizing xCT, a glutamate–cystine transporter (93) whose activity promotes cystine uptake for GSH synthesis (Figure 3). Other studies have shown that CD44-positive cancer cells up-regulate antioxidant genes (93, 94). In one gastrointestinal cancer mouse model, CD44-positive gastric tumor cells expressed a set of antioxidant genes at higher levels as compared with CD44-negative tumor cells (93). Furthermore, CD44 can attenuate activation of the mammalian Hippo, c-Jun N-terminal kinase, and p53 signaling pathways, resulting in resistance to oxidative and cytotoxic agent-induced stress in glioblastoma multiforme cells (95). These findings suggest that CD44 can maintain low levels of ROS and orchestrate protective mechanisms against ROS-mediated damage. Thus, CSCs and other CD44-expressing cancer cells may reduce oxidative stress through a variety of mechanisms.

### Resistance to antitumor immunity

CD44-expressing cancer cells can acquire the capability to evade immune destruction. For instance, the interaction of CD44 with HA fragments led to immune escape of lung cancer cells from cytotoxic T lymphocyte-mediated cytotoxicity (96). In their study, Yasuda et al. proposed a mechanism by which HA–CD44 interactions reduced the susceptibility of cancer cells to Fas/Fas ligand-mediated cytotoxicity by suppressing Fas expression. Moreover, the immunological properties of CD44-positive cancer stem-like cells have been compared with those of CD44-negative cells in squamous cell carcinoma of the head and neck. Unlike CD44-negative cells, the CD44-positive cells strongly inhibited T-cell proliferation and efficiently induced regulatory T cells (Tregs) and myeloid-derived suppressor cells. Interestingly, a model for how HMW–HA may play an important role in the maintenance of immunological tolerance has been proposed as well. HMW-HA provided co-stimulatory signals by cross-linking with CD44 and modulated the function of Tregs via production of IL-2, IL-10, and TGF-β (97). IL-10 production is a reported mechanism by which Tregs maintain immune tolerance (98). Tumor-derived HA fragments have also been shown to promote the development of immunosuppressive M2 macrophages by dynamically educating recruited blood monocytes (99). Our recent study demonstrated the preferential engagement of immunosuppressive M2 macrophages in an HA-rich stromal microenvironment (47). The presence of a large pericellular HA coat likely impaired immune surveillance due to steric hindrance. Taken together, CSCs have a higher malignant potential due to numerous escape mechanisms from immune attack that will necessitate the development of novel strategies to overcome.

## Conclusion

This review emphasizes the diverse roles of HA and its CD44 receptor on the stemness and survival of CSCs. Recent studies have enabled us to postulate reliable mechanisms by which HA functions in the regulation of CSC maintenance that may be partly dependent on the mesenchymal conversion of cancer cells. Growing evidence also indicates that CD44 is not only a common marker of CSCs but also plays a dominant role in maintaining their stemness. HA and CD44 enhance cell survival and confer resistance to chemotherapy and oxidative stress in CSCs as well.

Intense efforts are currently being directed toward the development of therapeutic strategies that selectively target CSCs. A more complete understanding of the mechanisms controlling how HA and CD44 mediate the stemness of CSCs will aid in the development of new treatment strategies to improve the outcome of cancer patients.

## Conflict of Interest Statement

The authors declare that the research was conducted in the absence of any commercial or financial relationships that could be construed as a potential conflict of interest.
